# Complexity, context and considerations for treatment in CFS/ME: children’s versus health professional’s conceptual model

**DOI:** 10.1186/1745-6215-16-S1-P21

**Published:** 2015-05-29

**Authors:** Roxanne Potgieter, Aarti Patel, Lucy Beasant, Kirstie L Haywood, Debbie Johnson, Alison Shaw, Esther Crawley

**Affiliations:** 1 School of Social and Community Medicine, University of Bristol, Bristol, UK; 2 Department of Psychology, University of Bath, Bath, UK; 3 Royal College of Nursing Research Institute, Warwick Medical School, University of Warwick, Coventry, UK

## Background

Paediatric CFS/ME is relatively common and disabling. However, little is known about which outcomes are important to children and the clinicians who treat them.

## Methods

We conducted semi structured interviews with young people with CFS/ME. Paediatric CFS/ME clinicians participated in focus groups or semi-structured interviews. Data was analysed thematically, descriptive accounts produced, and theoretical explanations developed.

## Results

Thirty young people with CFS/ME were interviewed (15 males, 15 females); mean age 13.2 years (SD 2.3), and 15 clinicians from a range of clinical disciplines and experience in paediatric CFS/ME (2 months to 25 years) of which 10 (67%) were female.

Both young people and clinicians identified similar key outcomes: symptoms, activity, social participation and emotional wellbeing influenced by management and contextual factors. Disrupted school attendance was the impact described most often by young people. Clinicians said that changing sleep was fundamental for improvement. Clinicians described problems with using school attendance as an outcome measure as it is often reduced during treatment and did not necessarily reflect a child's disability or whether they were coping. Young people described a unidirectional relationship of anxiety, low mood and stress as a consequence of their symptoms and the reduction in usual activities, socialising and ability to keep up with school. Clinicians revealed the circularity of low mood with children becoming more vigilant to symptoms and lower thresholds for activity and participation driving further low mood.

## Discussion

Analysis supported the development of separate conceptual models of CFS/ME described by young people and clinicians. Both the young person and health professional’s perspective is important for understanding the impact of this complex disabling illness.

